# Round the Hospitals

**Published:** 1918-10-26

**Authors:** 


### ROUND THE HOSPITALS.

The Queen has given £1,000 in response to the Red Cross "Our Day" appeal, "in grateful recognition of the work which the Joint Committee of the British Red Cross Society and the Order of St. John and those working under it have accomplished." Her Majesty adds: "During the past four years of war I have visited many hospitals in this country and in France, and everywhere I have found evidence of the anxiety of the Red Cross to further the efforts of the naval and military Services in their task of relieving the sufferings of the sick and wounded." Among the countless generous supporters of the Red Cross there is none, we believe, better qualified by knowledge, sympathy, and experience to form a judgment on its work than the Queen. Her continual interest in hospital work and presence among the sick and wounded constitutes at once a stimulus and a protection for which nurses and patients alike cannot be too grateful.

**ROUND THE HOSPITALS**—*(continued)*.

IN THE HOSPITAL of the 19th inst. we published a special account of a remarkable Day of good works which Queen Alexandra spent in Norwich, blessed by and blessing everyone; and they were a multitude, with whom H.M. was brought in contact. We have it on authority that it was a most successful day, and we are grateful and not surprised to learn that Queen Alexandra never had a more enthusiastic reception. Louisa Warnes, of blessed memory, would have rejoiced to see this day, and we confess to the conviction that she must have shared the enthusiasm and extended her blessing to the proceedings.

THE KING has been pleased to approve of the award of the Military Medal to the following nursing sisters for distinguished service in the field:—Sister Jane Elizabeth Trotter, Q.A.I.M.N.S.(R.), for gallantry and devotion to duty during an air raid which lasted from 11 P.M. till 3 A.M. Sister Trotter was in charge on night duty, and visited all the wards during the raid, reassuring the sick and wounded. Her orderly was mortally wounded whilst standing by her in one of the wards. Her conduct was most praiseworthy. Sister Ethel Frances Watkins, Q.A.I.M.N.S.(R.), for gallantry and devotion to duty during a raid which lasted four hours. She was wounded by a piece of shrapnel, but, with wonderful coolness, made light of the injury, and set a magnificent example. Staff Nurse Agnes Jack Parker, T.F.N.S., for gallantry and devotion to duty under trying conditions when heavily bombed by hostile aircraft at night. Her ward was badly damaged in the raid by a bomb falling close to it. By her exceptional coolness and complete disregard for her own safety she set a splendid example and gave great confidence and comfort to the patients. Such splendid deeds do more than rejoice the heart. They make little sacrifices of ease and comforts positively welcome to those whose lot is far from danger.

AT an Investiture held at Buckingham Palace on Saturday last the King conferred the decoration of the Royal Red Cross on the following: *First Class*.—Matrons Ethel Davidson and Clara Ross, and Head Sister Clarice Dickson, all of the Australian Army Nursing Service. *Second Class*.—Sisters Gertrude Thomas and Beatrice Thompson, of the Territorial Force Nursing Service; Head Sister Alice Douglas, Australian Army Nursing Service.

THE Matrons-in-Chief of the Military Nursing Services of the Crown, Dame Ethel Becher (Q.A.I.M.N.S.), Dame E. M. McCarthy (France), Miss Sidney Browne (T.F.N.S.), Miss Macdonald (C.N.S.), Miss Conyers (A.N.S.), and Miss Thurston (N.Z.N.S.) are to be entertained at luncheon on Wednesday, October 30, at the Trocadero Restaurant. The luncheon is being given by representative women in Government Departments, the professions, and the arts, and the committeee which has been formed to organise the arrangements is under the chairmanship of Mrs. Massey Lyon, of the *Queen*. Princess Louise, Duchess of Argyll, will preside, and women distinguished in many branches of public work will be present. Advantage will be taken of the occasion to present an address, by Mrs. Humphry Ward, of appreciation of the great work which has been done by the heads of the Nursing Services of the Empire.

ABOUT this time last year we called attention to the zeal of those matrons who made a practice of providing their institutions as far as possible with home-made jam and other food accessories. Oranges, quinces, and a host of garden produce were treated with success in various ways, and in some hospitals the result was to provide hundreds of pounds of jams and marmalade, dozens of bottles of fruit, a sufficiency of chutney and other pickles to last through the winter. This year, so far, we have had no communication from any hospital matron, though we have awaited some during the last fortnight with pleasant expectation. We should take it as a great kindness, for we regard as a matter of some importance the cultivation of hospital gardens, the preserving of fruit and other garden produce, the yield of potatoes and all kinds of vegetables, if our readers who have devoted time to their gardens and allotments would send us an account of the results which have attended thei efforts in the special difficulties which the year has produced, comparing results attained this year with last. It would be a further kindness, which the Editor would appreciate, if those matrons who are able would send him a sample pot of the marmalade and jam, a sample pumpkin, or a few potatoes, which they consider to be the best of their harvest this year. If this idea is welcome to our readers we shall hope to pursue it. It is requested that all samples may be accompanied by an invoice, so that those who have given their time to the work shall be saved all expense. Allotments have excited so much general interest since the war, that, with the aid of our readers, a full account of this side of war results, which has brought increased health and happiness to many, should prove welcome to all.

INFLUENZA is a perennial disturber of plans and time-tables in institutions, and just now is making worse havoc than ever with staffs already depleted to danger-point. The Edmonton Military Hospital has suffered acutely. Miss Michael, a probationer from Glasgow, and Miss Evans, one of the nurses from Carmarthen, have both died from infection caught during the performance of their duties, while nearly a dozen other nurses are ill from the same cause. The Passmore Edwards Hospital at Wood Green is closed, and all the nursing staff but three are ill in bed with influenza. Great watchfulness is indicated, and any rise of temperature should be treated by instant confinement to bed. The main danger is that of neglecting early symptoms.

WE regret to announce the death, on October 18, from influenza and pneumonia, of Miss Kathleen Sinclair Stewart, A.R.R.C., matron of the Welsh

**ROUND THE HOSPITALS—(*continued*).**

Hospital, Netley, to which office she was appointed only last June. Despite this short period of service at Netley Miss Stewart had already made her influence felt, and had won for herself the confidence of all with whom her work brought her in contact. She will be greatly mourned and missed. She was buried on October 23 at Blair Atholl, a memorial service being held in the Garrison Church at Netley.

In the capture of Damascus, one of the most enthralling incidents of the year, three Englishwomen—Miss Lawford, of the Church Missionary Society; Miss Johncock, of the Edinburgh Medical Mission; and Miss Basham, of the Jerusalem and the East Mission—were discovered peacefully serving in their captivity as nurses in the Victoria Hospital, which had been converted into a Red Crescent hospital. Miss Lawford, who was in temporary charge of the C.M.S. Girls' Orphanage at Nazareth when war broke out, bravely remained at her post in order to get the seventy orphans under her care to places of safety, but she and her charges were at length forced to leave by the Turkish soldiers. Nazareth became a great hospital base, and here, among her ministrations to the wounded, Miss Lawford had the happiness of tending some of our own officers and men. Last January she and the other two nurses were deported to help in Red Cross work at Damascus, where they were found and released. The Nazareth orphanage has done fine work in the past, and among the 400 girls it has trained many are now carrying on the torch as teachers and nurses. The number of war orphans is immense, and a great extension is called for.

Among the facts about "Our Work" circulated in connection with "Our Day" the Red Cross authorities note that they have 2,250 trained nurses working at home and abroad, with nearly 9,000 V.A.D.s helping in Navy and Army hospitals. It must considerably have surprised those armchair critics who accuse Devonshire House of incompetence because occasionally the demand for V.A.D. workers outstrips the supply to learn that Queen Mary's Army Auxiliary Corps, together with the Women's Royal Naval Service and the corresponding Women's Air Service, are calling out for "thousands" of fresh recruits. The truth is that as the war progresses the pace is continually accelerated, until every able-bodied woman, as well as every man, is required to bring about complete and conclusive victory. It is not by querulous complaints of existing organisations, all over-strained almost to breaking-point, that the day will be won.

The debate as we go to press in the House of Commons on the question of women's admission to Parliament is likely to be one of extraordinary interest. It is well known that many women take but a faint interest in the exercise of a vote which cannot be cast for one of their own sex, and the admission of a few women, doers rather than talkers, to the House of Commons would be a far more real advance than the bestowal of the franchise. As members of committees on matters affecting the welfare of women their presence is often urgently needed. It is interesting to recall that in Canada Miss McAdams, sister in the Canadian Military Hospital, was recently elected by Alberta soldiers serving overseas over the heads of fifty male candidates.

The General Committee of Queen Mary's Hospital at Stratford have made a substantial increase in the nurses' salaries. For the future probationers are to start at £12 a year, and rise in successive years to £35. Ward sisters are to have £45 to £60; night sisters £50 to £70; massage sister £60 to £70; out-patients' sister £50 to £70. Extra off-duty time and holidays are to be granted "when circumstances permit." The committee have also decided on a pension scheme for their nurses. Such as remain twenty-five years in the service of the hospital are to be assured £52 a year, dating from July 1, 1919, and members of the staff leaving on account of ill-health after ten years' service are to receive such pension or gratuity as the committee thinks fit. The scheme is non-contributory and open to criticism. A reserve fund must be accumulated to meet the possible demands for pensions, and it is doubtful whether, under the circumstances, and taking into account the facilities offered by the Royal National Pension Fund for Nurses, this is good finance.

It is a disputed question, one which some hospital committees have answered in the negative, whether it is wise to tie up nurses in their own pecuniary interest to stay as long as twenty-five years in one institution. Women are apt to get very stale under these conditions, unless they are of the rare stay-at-home type. Conditions of training alter, and the sister who has been trained twelve or fifteen years ago may retard progress. If she is lingering on to secure a pension she cannot in common justice be asked to retire, even if manifestly deteriorating. Again, the committee appears to be overlooking in the interests of a few stayers those of the younger women who are passing on. To stimulate nurses to open an account with the Royal National Pension Fund for Nurses by paying part premiums so long as they remain in the service of the hospital constitutes a real and far-reaching stimulus to thrift. Saving is a habit which needs to be taught. The offer of non-contributory pensions does little to inculcate it.

The West Ham Guardians have suddenly made the really astounding discovery that they are paying their scrubbers at a considerably higher rate than their staff nurses. This is what comes of beginning at the wrong end in revising rates of pay. The pay of the scrubbers works out at £1 16s. 6d. a week, or £94 18s. per annum, while that of the staff nurses, including uniform, is £82, equivalent to £1 11s. 6d. a week. In both cases 10s. a week is reckoned for rations. The executive officer in charge of a ward receives, as the chairman himself brought to notice, less than the utterly unskilled

**ROUND THE HOSPITALS—(*continued*).**

worker. The West Ham Guardians are justly proud of their infirmary, and have proved themselves by no means niggardly in their dealings with the needs of the sick poor. Their chairman is keenly interested in raising the status of nurses, and expressed his opinion that the nurses ought to rise to £150. But there cannot be two opinions about their parsimony in respect of their nurses' salaries. The probationer nurses of the board have now asked for an arbitration on the question of their salaries, and the Ministry of Labour is appointing an arbitrator for that purpose, and will receive five members of the West Ham Board as representatives. The award will be awaited with much interest, and is likely to have a far-reaching effect.

---

THE Reigate Guardians, being faced with an actual catastrophe in the infirmary from lack of nurses, have at length consented to increase the pay offered to the staff. The superintendent nurse is to have £80; charge nurses, £50; staff nurses, £35, £40; assistant nurses, £25, £30. The discreditable fact emerges that the Guardians have parsimoniously permitted for some time past the numerical strength of the staff to be reduced to an extent described as "lamentable." Not only has the well-being of the patients been seriously interfered with by this shortage of the staff, but the health of the few devoted women who have kept things going only by incredible exertions has been gravely imperilled. We should like to see some evidence of appreciation given to them in the shape of a solid bonus in return for all their off-duty time surrendered and the extra duties they have bravely, if not always quite uncomplainingly, performed.

---

THE authorities of the Royal Infirmary, Sheffield, have decided for the future to permit nurses with two years' fever training to enter the infirmary for three years instead of four to gain their certificates of general training. They make a similar concession for nursing members of a Voluntary Aid Detachment who have served for two years in a military hospital and given satisfaction. The matron, Miss Smeeton, is to be congratulated on this advance towards affiliation. The V.A.D. workers who desire to obtain general training are but a temporary factor in the situation. The fever nurses form a permanent and important branch of the nursing profession. It has been very discouraging for them to find their previous exact training in asepsis entirely ignored by matrons of the major training-schools, and we hope Sheffield is leading the way towards a better understanding.

---

THE last meeting of the Manchester Centre of the College of Nursing was held at the Ladywell Sanatorium, Pendleton, and broke new ground. Mrs. Rowan, lady-superintendent of the sanatorium, was hostess, and, having entertained the members at tea, she escorted them through several of the wards. The Children's Scarlet Fever Ward was empty and had been thoroughly fumigated, so members could linger and inspect its beautiful appointments before hearing a lecture from Dr. Mullen, the medical superintendent, on the feeding of fever patients, which was full of sound and helpful knowledge and greatly appreciated. The result was materially to lessen the unfortunate "isolation" which is apt to cling round the staff as well as round the finely constructed buildings dedicated to infectious patients. Much of the difficulty experienced in getting probationers for these institutions is the result of this isolation, and the Centre has done well in drawing the sanatorium within the magic circle of the College.

---

A BAZAAR in aid of the College of Nursing is to take place on November 5, 6 and 7 in Manchester, and on November 22 an address will be given to members on "The Responsibility of Women with regard to the Vote."

---

AMONG the immediate consequences of the German *débâcle* in France and Flanders has been the landing of crowds of German wounded on our shores, left behind by their comrades. The problem of giving these wounded prisoners adequate care has been at times singularly acute, and it is, of course, not one in the solution of which the public can be invited to share. All accounts agree that the German private is more often than not a decent fellow enough so far as submission to hospital regulations and gratitude for the ministrations he receives is concerned. But the officer, especially the Prussian officer, remains a cur. It will be for the German nation to detect in years to come and eradicate with all the resources at their command the deadly taint in their training which has poisoned the springs of thought and action in these men and reduced them below the condition of beasts of prey.

---

AMIDST the general shortage of hospital beds which accentuates this feverish moment in the history of the war, it is satisfactory to learn that the Brigade Hospital of the Order of St. John, which was so cruelly shattered by enemy bombs at Etaples, has been re-erected in another French town, and is already receiving patients. Although constructed in an incredibly short space of time, the new hospital is a marvel of comfort and scientific development in the interests of the patients and nursing sisters.

---

☛ **It is our invariable practice to pay for all items of news which we may publish. The minimum payment is 5s. Paragraphs of about twenty lines in length—that is to say, of 150 words or less—are preferred. Contributions should be addressed to The Editor, THE HOSPITAL, 28 and 29 Southampton Street, Strand, London, W.C. 2, and, to be in time for the current week's issue, should reach him by the first post on Mondays.**

